# Accurate diagnosis of mismatch repair deficiency in colorectal cancer using high-quality DNA samples from cultured stem cells

**DOI:** 10.18632/oncotarget.26495

**Published:** 2018-12-25

**Authors:** Tadayoshi Yamaura, Hiroyuki Miyoshi, Hisatsugu Maekawa, Tomonori Morimoto, Takehito Yamamoto, Fumihiko Kakizaki, Koichiro Higasa, Kenji Kawada, Fumihiko Matsuda, Yoshiharu Sakai, M. Mark Taketo

**Affiliations:** ^1^ Division of Experimental Therapeutics, Graduate School of Medicine, Kyoto University, Yoshida-Konoé-cho, Sakyo-ku, Kyoto 606-8501, Japan; ^2^ Department of Surgery, Graduate School of Medicine, Kyoto University, Shogoin-Kawahara-cho, Sakyo-ku, Kyoto 606-8507, Japan; ^3^ Center of Genomic Medicine, Graduate School of Medicine, Kyoto University, Shogoin-Kawahara-cho, Sakyo-ku, Kyoto 606-8507, Japan; ^4^ Office of Society-Academia Collaboration for Innovation, Kyoto University, Yoshida-Honmachi, Sakyo-ku, Kyoto 606-8501, Japan; ^5^ Present address: Department of Genome Analysis, Institute of Biomedical Science, Kansai Medical University, Hirakata, Osaka 573-1010, Japan

**Keywords:** colorectal cancer, spheroid, cancer stem cell, molecular oncology, immunotherapy

## Abstract

Mismatch repair (MMR)-deficient or microsatellite instability (MSI) colorectal cancer includes two subtypes; Lynch syndrome and sporadic MSI cancer, both of which generate multiple neoantigens due to unrepaired mutations. Although such patients respond very well to immune checkpoint therapy, their diagnosis can be confused by low quality DNA samples owing to formalin fixation and/or low cancer cell content. Here we prepared high-quality DNA samples from *in vitro*-cultured cancer spheroids that consisted of the pure cell population. We evaluated their diagnostic power by on-chip electrophoresis, mutational burden assessment, and direct sequencing. Because formalin-fixed paraffin-embedded (FFPE) tissues are widely used as the DNA source, we compared such samples with spheroid DNA. Additionally, we performed immunohistochemistry (IHC) for MMR proteins on spheroids as well as primary tumor sections. Of 111 cases of colorectal cancer patients, we found seven MSI-high cases in which all diagnostic results agreed on spheroid-based assays, whereas the results with the FFPE DNA were less reliable though analyzable. Importantly, there was an MSS case that appeared as MSI by IHC on primary tumor sections. Based on these results, we propose to employ cultured cancer spheroids as the source of both DNA and IHC specimens for more reliable clinical diagnosis.

## INTRODUCTION

Microsatellite instability (MSI) colorectal cancer is a hypermutable subclass due to mismatch repair (MMR)-deficiency. It includes two types; Lynch syndrome (LS) caused by germline mutations in the MMR protein genes, and sporadic disease without hereditary background but with somatic mutations and/or epigenetic changes in the responsible gene [1]. These mutations result from unrepaired replication errors including repetitive DNA sequences, causing short insertion-deletion (indel) mutations in mono- and di-nucleotide repeats, as well as other types of mutations [2, 3]. Therefore, MMR-deficient colorectal cancer subclass gives a strong indication for immune checkpoint therapy using anti–PD-1 or –PD-L1 antibodies, with remarkable clinical efficacy [4].

While this subclass takes up 5–15% of colorectal cancer cases, this proportion can vary not only among geographic populations, but also depending on the accuracy of diagnostic methods [5]. Currently, two major methods are employed. One is immunohistochemistry (IHC) of MMR proteins as adopted by many institutions as the standard test [5, 6]. It aims to detect lack of MMR protein(s) as MLH1, MSH2, MSH6, and/or PMS2 [7, 8], which can be misleading partly because the diagnosis depends on ‘loss’ of staining in cancer cells among co-existing stromal cells that show normal levels of expression.

The other is based on the analyses of tumor genome that shows microsatellite instability (MSI), increased mutational burden and mononucleotide repeat frameshift mutations in some key coding genes [9, 10]. In these DNA analyses, tumor DNA is usually extracted from either frozen or, more commonly, formalin-fixed paraffin-embedded (FFPE) tumor tissues resected surgically [11]. While laser-capture microdissection of frozen or FFPE samples can isolate cancer cell-enriched areas, this technique is labor-intensive, and may not be suitable for clinical screening service. In practice, macrodissection of cancer cells is widely used [4]. However, the purity of cancer DNA thus isolated can be compromised because the tumor tissues contain not only cancer epithelial cells, but also non-cancer stromal cells of which wild-type DNA may confuse the diagnosis [12]. Furthermore, DNA samples from FFPE tissues are chemically damaged significantly, often making the analysis difficult.

To overcome this problem of mixed cell population that composes the cancer tissue, it would be ideal if we can isolate only the cancer epithelial cells. To this end, it has become possible recently to culture and propagate the tumor-initiating cells (TICs, or cancer stem cells) in 3D matrix *in vitro* [13, 14]. This technique has provided not only cancer- but also normal epithelial-stem cells under rapidly growing conditions. Besides, it eliminates the stromal cells in the culturing process. Our current success rate for spheroid establishment in cancer- and normal epithelial-stem cells are ∼90% and 100%, respectively [14]. Because the majority of colorectal cancer patients undergo resection operations of the primary tumors, fresh tissue samples are usually available except for very early or inoperable late stages. Cancer spheroid cultures offer us excellent test materials for genomic and expression analyses as well as for immunohistochemical staining of MMR proteins. Furthermore, we have just reported that chemosensitivity of spheroid-derived xenografts accurately reflects that of the clinical response [15]. To prepare for prospective studies, we are currently culturing colorectal cancer spheroids of all resected stage III/IV primary tumors at Kyoto University Hospital. This particular report shows one of these efforts that take the advantage of available spheroid cultures. In the present study, we have investigated the feasibility and reliability of exploiting cultured spheroids as the source material for MSI diagnosis.

## RESULTS

### Detection of microsatellite instability (MSI) in colorectal cancer using on-chip electrophoresis of satellite marker PCR products from spheroid-derived DNA

We recently established spheroid cultures of colorectal cancer tumor-initiating cells (TICs, or cancer stem cells) as well as those of their normal epithelial stem cells from 111 specimens surgically resected at Kyoto University Hospital according to the protocol reported previously [13]. Clinicopathological characteristics of the patients are summarized in Table 1.

**Table 1:** Clinicopathological characteristics of patients

		*n* = 110^a^
Sex	Male	65
	Female	45
Age, years	Median (MIN–MAX)	69 (36–89)
Amsterdam criteria		0
Bethesda criteria		0
Past history	Lynch associated cancers of the colon, rectum stomach, uterine endometrium, small bowel and/or urinary tract	10
	Familial adenomas polyposis	0
Tumor location	Right side	38
	Left side	43
	Rectum	30
Tumor stage	1	11
	2	48
	3	36
	4	16
Histological grade	Low grade	102
	High grade	4
	Mucinous	5
No. of passages from spheroid establishment to extract DNA samples		
	Median (MIN–MAX)	5 (1–22)

^a^ One patient had synchronous double cancer in sigmoid and transverse colon.

**Figure 1:**
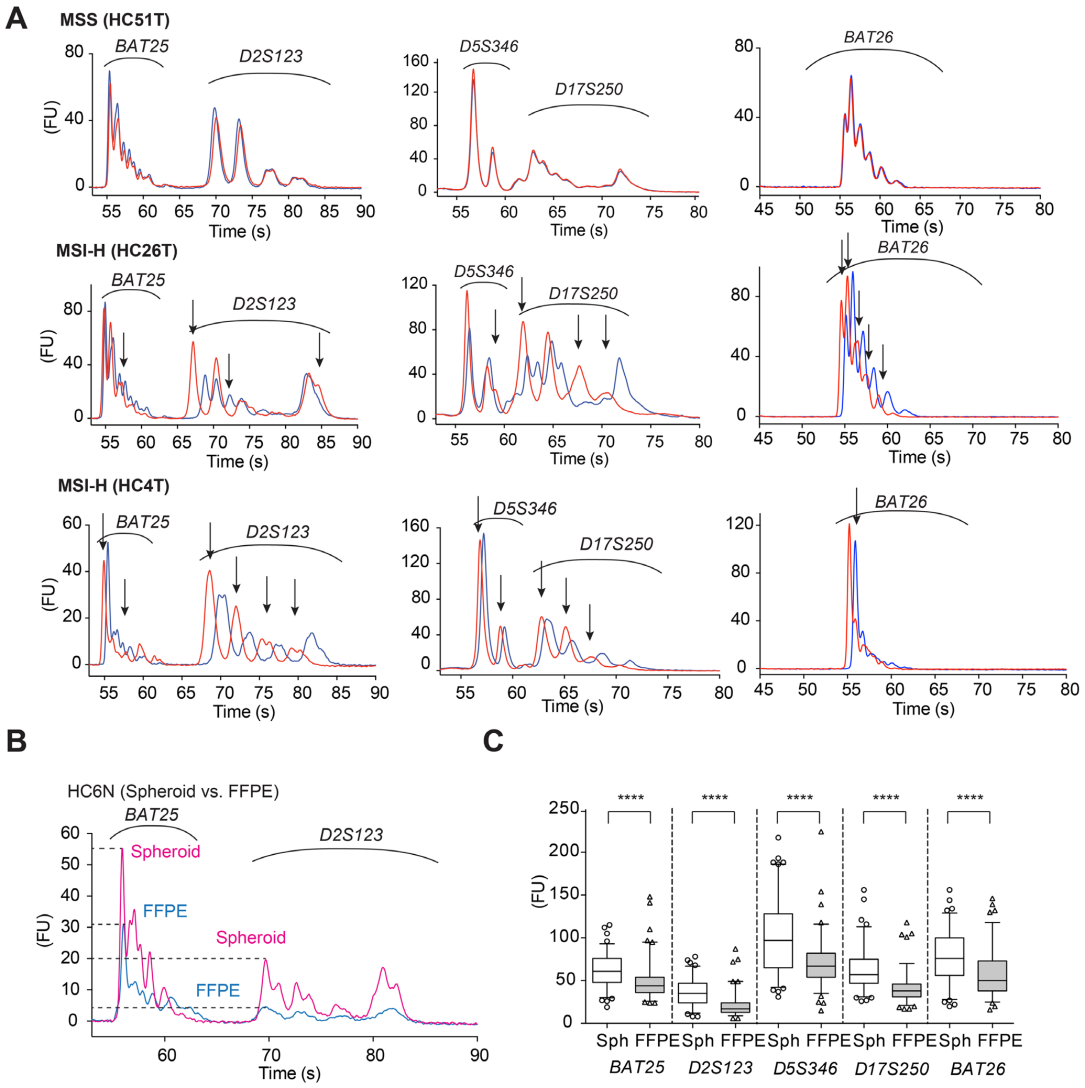
Electropherograms of on-chip MSI analysis using spheroid-derived DNA samples (**A**) HC51T. Representative electropherograms of an MSS case for five microsatellite markers of the Bethesda panel. Red lines show the PCR products amplified from the cancer cell spheroid DNA samples, whereas blue lines indicate those from the normal epithelial stem cell DNA of the same patient. HC26T and HC4T. Representative electropherograms of MSI-H cases. The peak patterns between tumor-initiating cells (red) and the normal epithelial stem cells (blue) are separated for all loci tested. The ordinate shows fluorescence intensity in arbitrary unit (FU). (**B**) On-chip electropherogram of a representative case in which FFPE tumor-derived DNA sample (blue) showed much lower peaks with poorer resolution than spheroid-derived DNA (red) of the same patient normal mucosal stem cells (HC6N). The ordinate shows fluorescence intensity in arbitrary unit (FU). (**C**) Maximum electrophoretic peak-heights of PCR-amplified MSI markers (shown at bottom) compared between spheroid- and FFPE tumor-derived DNA samples. Note that spheroid-derived (Sph) DNA gave taller peaks than FFPE tumor-derived (FFPE) DNA for all five Bethesda panel markers. ^****^P < 0.0001, two-tailed Mann-Whitney *U* test (*n* = 99).

To improve molecular diagnosis of microsatellite instability (MSI) colorectal cancer, we employed an on-chip MSI test [16], and examined the above 111 cases using DNA samples purified from cultured spheroid cells. To this end, we first extracted DNA from both cancer and normal epithelial spheroids of the same patients, and amplified by PCR five MMR-target microsatellite markers recommended in the Bethesda panel [1]; *BAT25, BAT26, D2S123, D5S346,* and *D17S250*. We then analyzed the PCR products by microfluidics-based on-chip electrophoresis in an Agilent Bioanalyzer (see Materials and Methods), and compared their electropherogram overlays regarding the peak positions between the cancer and normal cell DNA. This on-chip assay provides a higher resolution because of the shorter running time than the conventional fluorochrome-based assay (i.e., capillary electrophoresis) widely used in MSI test [16]. As indel mutations generated by DNA strand slippage during replication remained unrepaired in the genomic DNA of MMR-deficient cancer cells, the peak positions of their electropherograms often shifted from those of the normal epithelial cell DNA of the same patient (Figure 1A, HC26T and HC4T). In microsatellite-stable (MSS) cancer cells, the peak positions of the PCR products were identical between cancer and normal cells, as expected (Figure 1A, HC51T). We also show better resolution and lower noise obtained by the on-chip MSI electrophoresis of spheroid DNA in Figure 1A. For all satellite markers tested, the peak heights of PCR products amplified from spheroid-derived DNA were significantly higher than those of FFPE tissue-derived DNA (Figure 1B and 1C), which helped identify the difference between cancer and normal cell peaks.

We diagnosed the MSI status according to the Bethesda criteria that recommended calling MSI-high (MSI-H) when more than 30% of cancer cell microsatellite markers showed different peaks from normal cell markers [2]. Namely, in the set of five markers above, we designated MSI-H when two or more had shifted cancer peaks (e.g., Figure 1A, HC26T and HC4T). When only dinucleotide repeat markers as *D2S123*, *D5S346* and/or *D17S250* were mutated, a secondary panel of markers with mononucleotide repeats (*BAT40* and *MYCL*) was tested as recommended by the revised Bethesda criteria [1]. In some exceptional cases such as HC106T, only one marker (*D2S123*) showed clear shifts on peaks that reminded us of typical indel mutations. Accordingly we then tested two more satellite markers, and finally diagnosed the case as MSI-H (Supplementary Figure 1). Based on these results, we detected total of seven (6.3%) MSI-H cases and 104 MSI-L or MSS cases (30 MSI-L and 74 MSS).

### Mutational burden assessed by next generation sequencing (NGS) of spheroid-derived DNA

In MMR-deficient colorectal cancer, it is known that genomic mutational burden increases not only because of dinucleotide and mononucleotide repeats described above, but also due to increased mutations of various kinds [17, 18]. One of the methods to assess such a condition is to estimate the density of mutations. It has been reported that the whole genome mutational burden can be estimated in coding sequence subsets [19–21]. Thus, we sequenced the coding regions of 409 cancer-related genes spanning 1.29 Mb using cancer spheroid DNA samples (see Materials and Methods).

As described in Materials and Methods, however, this analysis was not so simple practically as it appeared in the concept. Although it was ideal to use spheroid DNA from the matched normal colonic epithelial cells of the same patients as references for cancer cells, this doubled the sequencing cost. As a more cost-efficient alternative, we referred to a database and eliminated polymorphic variants commonly found in the geographic population (see Materials and Methods, and Supplementary Figure 2). Just in case, we also sequenced the matched normal epithelial spheroid DNA for two MSI-H cases, and used the data as additional references. These results were similar to those obtained by the alternative method using a normal population variant database (Supplementary Figure 3).

**Figure 2:**
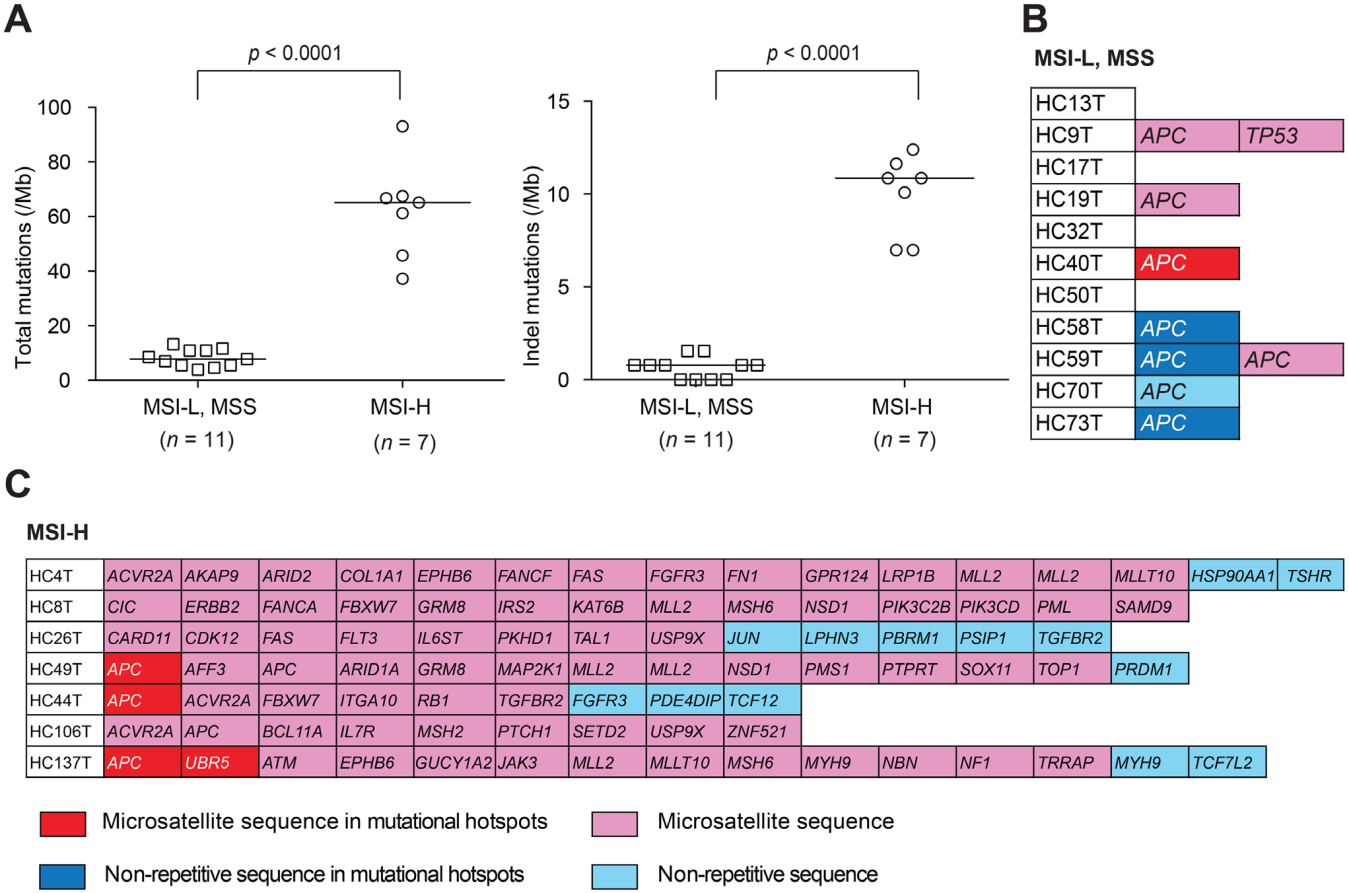
Mutational burden estimation (**A**) Total (left) and indel (right) mutational densities of MSI-H colorectal cancer spheroid DNA samples compared with those of MSI-L or MSS spheroid determined by NGS for 409 cancer-related genes spanning 1.29 Mb of the coding sequences. (**B**, **C**) List of indel mutations detected in the coding regions of 409 cancer-related genes in 11 MSI-L or MSS colorectal cancer spheroid DNA samples (B) and in seven MSI-H cases (C). Color codes in (B, C) indicate the locations of mutations: Red, in microsatellite sequences of mutational hotspots; Pink, in microsatellite sequences; Dark blue, in non-repetitive sequences of mutational hotspots; Light blue, in non-repetitive sequences.

Using the variant-filtering method (Supplementary Figure 2), we searched for cancer-driving mutations in seven MSI-H and 11 MSI-L/MSS cases (Supplementary Tables 1 and 2, respectively). Consistent with an earlier report [4], the results showed significantly higher mutational densities in the MSI-H tumor genome than in the MSS, regarding both total mutations (median, 65 vs. 8; *p* < 0.0001) and indel mutations (median, 11 vs. 1; *p* < 0.0001) (Figure 2A, left and right, respectively). Specific indel mutations found in the sequenced genes are shown in Figure 2B and 2C. In the MSI-L or MSS cases, mutations of tumor DNA were found typically in *APC* and/or *TP53* whereas, in MSI-H tumors, they were in a variety of cancer-related genes.

In addition, we sequenced FFPE cancer tissue-derived DNA from two MSI-H cases, HC4T and HC49T for 409 cancer related genes (Supplementary Figure 4). The quality of the FFPE tumor DNA data were significantly lower than those of spheroid DNA, although the overall numbers of mutations/variants appeared similar. For example, in colon cancer HC4T, we found 12 of 79 variants were specific to FFEP DNA in addition to 67 common variants including three key mutations (in *BRAF, MTOR* and *RUNX1*; Supplementary Figure 4). However, the allelic frequencies for 11 of the 12 were < 40%. Such low frequencies were likely caused by the co-existing stromal cells in the FFPE tumor tissues. This is in a strong contrast to 20 (23%) variants that were specific to the spheroid DNA of which allelic frequencies for 16 of the 20 variants were higher than 40%, suggesting that they represent the majority of cancer cells. Accordingly, the same ambiguity can remain even if frozen cancer tissues are used as the DNA source instead of FFEP tumors. In this regard, some recent studies reported poor tumor cell purity in cancer tissue samples [12, 22, 23], one of which demonstrated low tumor cell contents that reduced the mutation frequency for key cancer mutations to < 5% [12]. Another study reported the discordance of sequencing data between cultured patient-derived cancer cells and primary tumors [23]. The low tumor purity samples reduced mutational frequency below the variant-calling limit in high-throughput DNA sequencing. Therefore, it appears that the difference in the DNA source can affect the accuracy of cancer diagnosis significantly.

### Direct sequencing of spheroid-derived DNA for MMR-target key coding genes

While mutations in the non-coding satellite markers of MMR targets can be detected by on-chip electrophoresis, most key functional targets that are affected in cancer by MMR deficiency reside in mononucleotide repeats in the coding sequences, often leaving frameshift mutations unrepaired in MSI-H cancer cells [5, 18]. To investigate whether above on-chip electrophoresis results were consistent with the mutational status in such MMR targets, we selected four representative genes; *TGFBR2* (A_10_), *BAX* (G_8_), *IGF2R* (G_8_), and *CASP5* (A_10_). After amplifications by PCR on cancer spheroid DNA, we directly sequenced the products for mutations in the mononucleotide repeats (see Materials and Methods).

**Figure 3:**
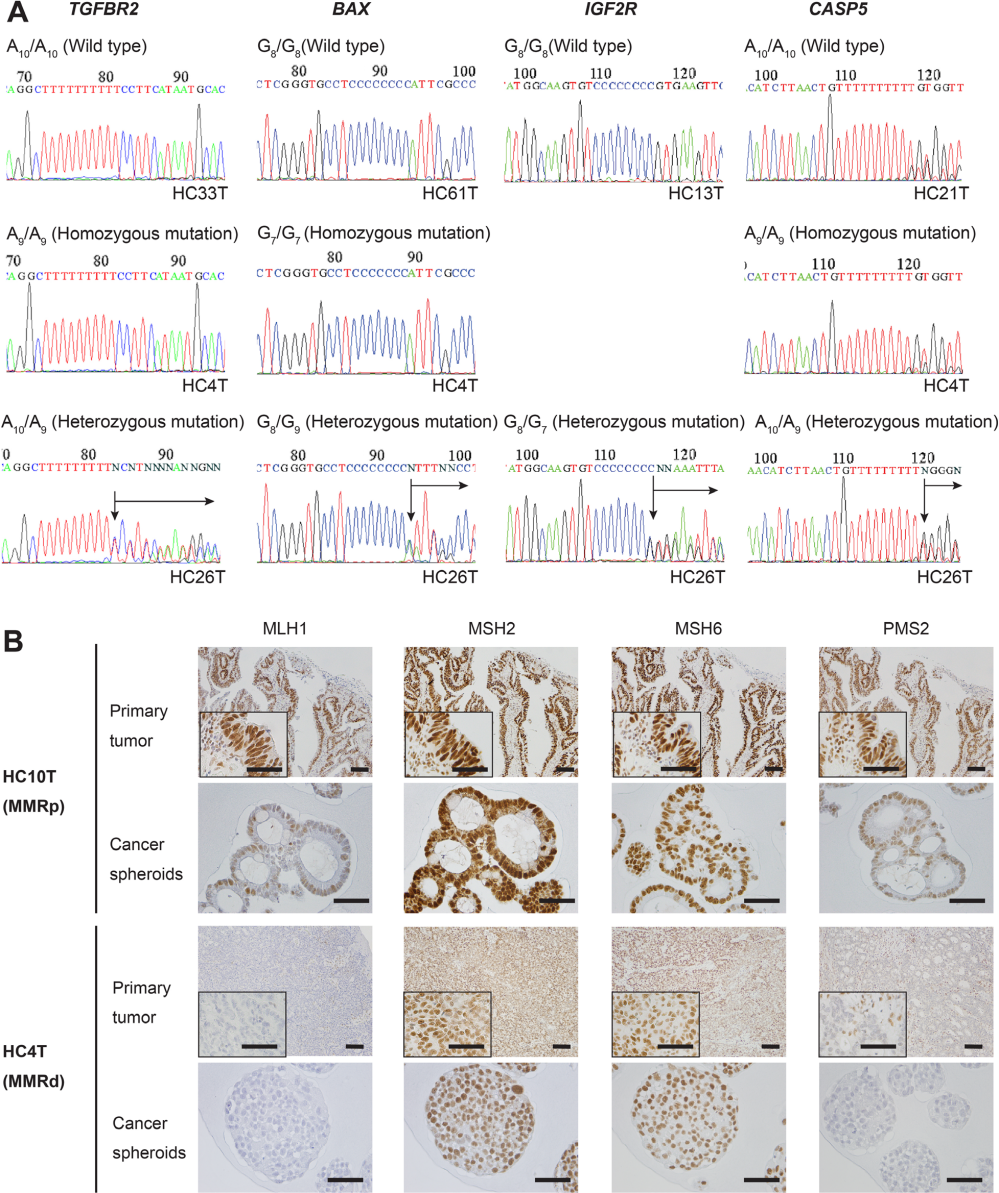
DNA sequencing analysis of some MMR-target coding mononucleotide repeats, and IHC for MMR proteins in primary colorectal cancer tissues or spheroid samples in MSI-H and MSI-L/MSS colorectal cancer cases (**A**) DNA sequencing data that cover the mononucleotide repeat regions amplified from the spheroid-derived DNA samples for *TGFBR2*, *BAX*, *IGF2R, and CASP5*. For all markers, the reverse strands of the wild types (top panels), and homozygous (middle) and heterozygous (bottom) mutants are shown. No cases were found with *IGF2R* homozygous mutation. (**B**) Immunohistochemistry for mismatch repair (MMR) proteins, MLH1, MSH2, MSH6, and PMS2. The colorectal cancer (CRC) HC10T (top panels) shows a MMR-proficient (MMRp) example of the primary CRC section (Primary tumor), with expression of all MMR proteins. The CRC HC4T (bottom panels) shows a MMR-deficient (MMRd) case. Expression of MLH1 and PMS2 was not detected in the primary tumor tissue. Notably, IHC of spheroid cells derived from the same tumors mirrored the results with the whole tumor sections. Note that some staining of MLH1 and PMS2 in HC4T primary CRC sections is in the stromal cell nuclei.

Because of the high purity of spheroid DNA both chemically and cell population-wise, it allowed us to detect not only homozygous but also heterozygous mutations in the MMR-target repeats by Sanger sequencing of the PCR products. For example, A_10_/A_10_ of *TGFBR2* mutated into A_10_/A_9_ is ending as an A/G superimposed peak (Figure 3A, *TGFBR2*). Beyond this position, all nucleotide peaks of the wild-type DNA strand superimposed with those of the mutated strand down to the first frameshift termination codon. Other representative sequence data for *BAX*, *IGF2R* and *CASP5* are also shown (Figure 3A). We noted that these mutations were often eliminated by variant calling software although they had remained in the raw data (Supplementary Figure 2).

**Figure 4:**
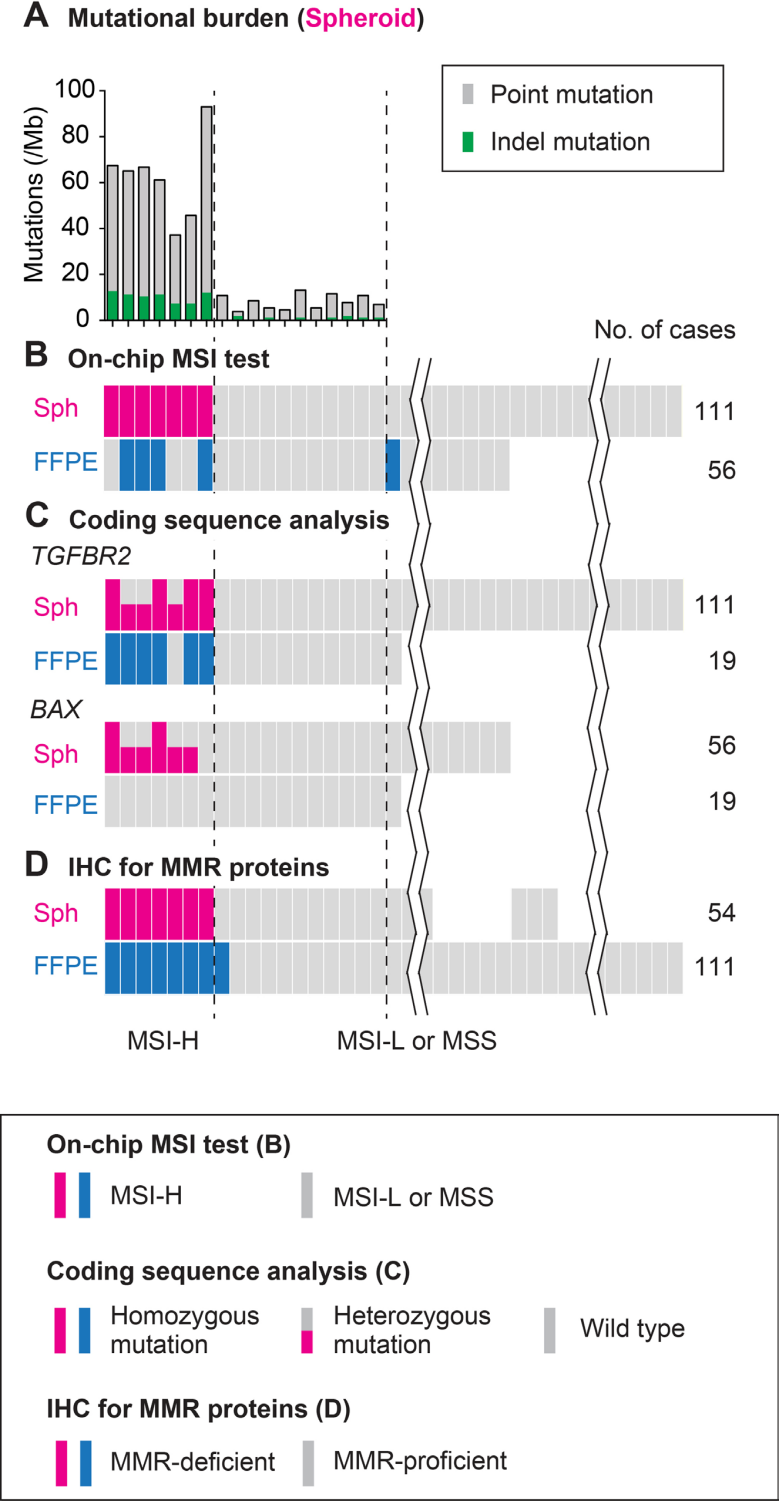
Schematic summary of the analysis results using colorectal cancer spheroids compared with those using FFPE tumors (**A**) Mutational burden estimated by exonic sequencing of spheroid DNA for 409 cancer related genes. (**B**) MSI status judged by on-chip electrophoresis of the Bethesda panel markers. (**C**) Mutations in the mononucleotide repeats in coding regions of *TGFBR2* and *BAX* determined using spheroid and FFPE tumor DNA. (**D**) IHC results of the cultured spheroids of tumor-initiating cells and FFPE primary tumors. Color keys are shown in boxes. See text for details.

By this method, we detected frameshift mutations in all seven MSI-H cases (100%) regarding *TGFBR2,* whereas in six cases (86%) for *BAX* and *CASP5,* and in three (43%) for *IGF2R*. Notably, we did not detect such mutations in any of the MSI-L or MSS spheroids regarding *TGFBR2* (104 cases tested) or *BAX* (49 cases tested) (Figure 4). These results strongly suggest that our on-chip electrophoresis of microsatellites was as accurate as direct sequencing of the key MMR-target coding genes (see below).

### Immunohistochemistry (IHC) for mismatch repair proteins on FFPE tumor tissues and on cancer spheroids

For clinical diagnosis of MMR-deficient colorectal cancer, it is common to detect loss of MMR proteins in tumor tissue sections by immunohistochemistry (IHC). To investigate how tight the correlation is between the MSI status determined using spheroid DNA and that by MMR protein expression, we next performed immunohistochemistry (IHC) on four MMR proteins, MLH1, MSH2, MSH6, and PMS2 [7] in the primary tumor sections of all 111 cases. Some staining data for the representative cases are shown (Figure 3B). As the result, we detected eight MMR-deficient cases and 103 MMR-proficient ones. Of seven MSI cases, five cases lost both MLH1 and PMS2 protein expression, and were assumed as sporadic cancer [25]. Remaining one case was confirmed for loss of MSH6 expression (HC137T), whereas the other for loss of PMS2 (HC8T), both of which were suspected of Lynch syndrome (Table 2). However we did not pursue further diagnostic tests. Notably, an MSS case with low mutational burden in spheroid DNA genomic analyses appeared as MMR-deficient by IHC (see HC13T below), although this difference of one in 111 cases was too small for statistical significance.

**Table 2:** MSI-H colon cancer cases assessed by (1) on-chip electrophoresis of five satellite markers, (2) DNA sequencing of four exoninc MMR deficiency target mononucleotide repeats, and (3) IHC for four MMR proteins MLH1, MSH2, MSH6 and PMS2

	On-chip		Sequencing of MMR deficiency target genes^c^				IHC	
Case	MSI status^a^ (Sph^b^)	MSI status^a^ (FFPE)	*TGFBR2* (WT: A_10_)	*IGF2R* (WT: G_8_)	*BAX* (WT: G_8_)	*CASP5* (WT: A_10_)	Absent proteins^d^ (Sph^b^)	Absent proteins^d^ (Primary)
HC4T	H	L	A_9_/A_9_	WT/G_7_	G_7_/G_7_	A_9_/A_9_	MLH1, PMS2	MLH1, PMS2
HC8T^e^	H	H	WT/A_9_	WT/G_9_	WT/G_7_	A_9_/A_9_	PMS2^f^	PMS2^f^
HC26T	H	H	WT/A_9_	WT/G_7_	WT/G_9_	WT/A_9_	MLH1, PMS2	MLH1, PMS2
HC49T	H	H	A_9_/A_9_	WT/WT	G_7_/G_7_	WT/A_9_	MLH1, PMS2	MLH1, PMS2
HC44T	H	L	WT/A_11_	WT/WT	WT/G_9_	WT/A_9_	MLH1, PMS2	MLH1, PMS2
HC106T	H	L	A_8_/A_9_	WT/WT	WT/G_9_	WT/WT	MLH1, PMS2	MLH1, PMS2
HC137T	H	H	A_9_/A_9_	WT/WT	WT/WT	A_8_/A_9_	MSH6	MSH6
	**Mutation frequency**		100%	43%	86%	86%		

^a^H: MSI-High, L: MSI-Low

^b^Spheroid

^c^Only spheroid DNA data were collected.

^d^Absent MMR proteins in immunohistochemical staining

^e^*MLH1* V384D mutation (see Discussion, Results and Supplementary Figure 7)

^f^*PMS2* R563X mutation

Colon cancer HC13T was diagnosed as MSS by three genomic analysis methods (on-chip MSI test, mutational burden assessment, and four MMR target mononucleotide repeats) using spheroid DNA derived from two independent clones as well as one FFPE tumor-derived DNA (another DNA sample showed MSI-L) (Supplementary Figure 5). On the other hand, IHC of cancer spheroid cells of two independent spheroid clones demonstrated contradictory results; one MMR-proficient with all four MMR proteins, and the other without MLH1 and PMS2 (Supplementary Figure 6A). Accordingly, we examined 14 sub-legions of the primary tumor for MLH1, MSH6 and PMS2. As the results, IHC for 7 of 13 sub-lesions were evaluated as MMR-deficient because both MLH1 and PMS2 appeared missing (Supplementary Figure 6B, 6C and Supplementary Table 3). Regarding this discrepancy between the DNA and IHC data, it is worth noting that the patient was treated with oxaliplatin before the surgery. It has been reported that platinum-based chemotherapeutics can affect expression of MLH1 and MSH2 in IHC [26], and induce the methylation of *MLH1* gene in cancer cells [27]. These results suggested that lack of MLH1 and PMS2 staining in this particular tumor was caused by platinum chemotherapy. Because this took place shortly before the surgery, it is reasonable to speculate that lack of MMR proteins did not accumulate mutations significantly in the whole genome. Accordingly, we concluded that the tumor HC13T was originally MSS, and that the MSI phenotype with IHC was caused by oxaliplatin neoadjuvant therapy. Thus, this particular cancer was not expected to contain enough neoantigens to respond to immune therapies.

It is worth noting that MSI tumor HC8T (Table 2) lacked PMS2 staining and had heterozygous R563X mutation (Supplementary Figure 7). Interestingly, the tumor was immunostained for MLH1, and contained variant mutation V384D [28]. As reported, the MMR phenotype of MLH1 V384D mutation can vary [28]. Here, we found four cases of V384D mutant tumor that ranged from MSI-H (HC8T above) to MSI-L (HC20T; a double cancer with HC8T) or MSS (HC25T and HC34T; Supplementary Figure 7). In IHC of both the primary tumor and spheroid samples, MLH1 immunostaining was absent (–) in HC25T, moderate in HC8T (+) as described above, and positive (++) in HC20T and HC34T. Importantly, immunostaining for PMS2 that forms a complex with MLH1 [8] was positive in all these cases except HC8T that was MSI-high. It is conceivable that MLH1 V384D mutation affected its antigenic epitope detected by the antibody used here and/or its interaction with PMS2.

Altogether, these results of the four methods led us to conclude that seven of 111 cases (6.3%) were diagnosed as MSI-H, although eight were MMR-deficient as described above. As reported earlier, IHC may cause difficulty in diagnosis [29] and spheroid DNA analysis can provide more accurate diagnosis (Figure 4, and Table 2).

### Spheroids provide better quality DNA more suitable for MSI diagnosis than FFPE-tumors

**Figure 5:**
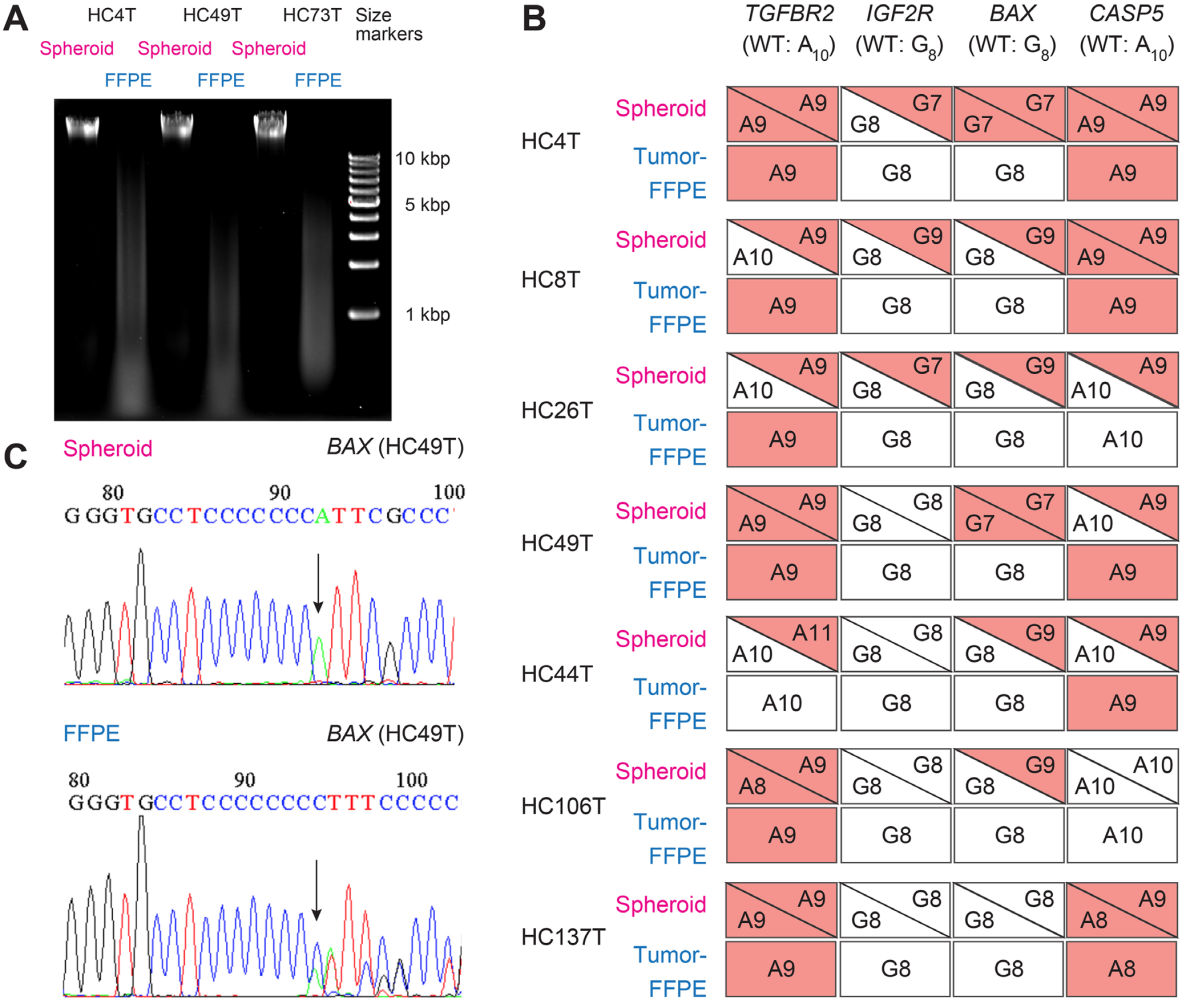
Comparison of cancer spheroid- and FFPE tumor-derived DNA samples in sequence analysis of MMR-target coding mononucleotide repeats (**A**) Agarose gel profile comparing the DNA samples from spheroid and FFPE tumors. (**B**) Mutation profiles of coding mononucleotides in four MMR-target genes for seven MSI cases analyzed with spheroid- and FFPE tissue-derived DNA samples. Red boxes/triangles indicate mutated alleles, whereas white ones show the wild-type. Analysis with spheroid-derived DNA enabled unambiguous identification of both alleles. However, FFPE tissue-derived DNA often gave confusing results. (**C**, top) An example of homozygous mononucleotide repeat mutation in *BAX* (G8 → G7), detected using the spheroid-derived DNA. Arrow points 8th G → T (C → A in reverse strand sequenced here). (bottom) With the FFPE tissue-derived DNA, a slightly low peak for the 8th G was detected after seven G peaks (C in reverse strand here). This is likely derived from DNA of the normal (i.e., wild-type) stromal cells rather than cancer epithelial cells.

In medical research as well as in clinical services, DNA analyses are often performed on tumor DNA extracted from formalin-fixed paraffin-embedded (FFPE) tissues. To evaluate the quality of DNA samples derived from FFPE tumors compared with that from colorectal cancer spheroids, we performed on-chip MSI tests using DNA from both sources for 50 tumors. To this end, we first assessed the chemical purity of DNA samples obtained from spheroids and FFPE tumors by reading the A260/A280 ratio. They were 1.8 and 1.7, respectively, both showing satisfactory purity for further analyses though the difference was statistically significant. We then estimated the DNA chain length by agarose gel electrophoresis. Compared with the spheroid-derived DNA samples, the FFPE tumor DNA showed smears trailing into smaller chain lengths, indicating substantial fragmentation due to chemical damages by formalin exposure (Figure 5A).

We next tested the five satellite markers by on-chip electrophoresis. With the spheroid-derived DNA samples of 50 tumors, we diagnosed five cases (10%) as MSI-H, and 45 cases as MSI-L or MSS. With the FFPE-tumor DNA, on the other hand, we detected only three (6.0%) MSI-H cases, misdiagnosing two MSI-H cases as MSI-L, whereas one MSS as MSI-H. Namely, two cases (HC44T and HC4T) were called as MSI-L with FFPE tumor DNA despite that they were MSI-H with the spheroid DNA. By adding the results of two more satellite markers (i.e., *BAT40* and *MYCL1*) in the on-chip electrophoresis, both samples showed more than 30% (i.e., three of seven) unstable markers even with the FFPE tumor DNA. The results of direct sequencing analysis of spheroid DNA for four mononucleotide-repeat markers (i.e., *TGFBR2, IGF2R, BAX,* and *CASP5*) confirmed their final diagnosis as MSI-H (Supplementary Figure 8, Figure 5B, and Supplementary Table 3). These additional assessments were performed to resolve discrepant MSI status resulted from the data with spheroid and FFPE DNA samples. Thus, analysis on spheroid-derived DNA allowed us to promptly detect all MSI-H cases according to the Bethesda criteria with five markers (1).

On the other hand, case HC24T was diagnosed as MSI-H with the FFPE tumor DNA by on-chip electrophoresis of the five marker-panel despite it was MSS with the spheroid-derived DNA (Supplementary Figure 9A and 9B, FFPE1). Upon sequencing of spheroid DNA for the four MMR-target mononucleotide repeats, all markers were homozygous wild type (Supplementary Table 3). Interestingly, when we analyzed by on-chip MSI test the FFPE tumor DNA samples extracted from two more separate subregions of the HC24T tumor, both turned out as MSI-low or MSS (Supplementary Figure 9B, FFPE2 and 3). Thus, we diagnosed this particular case as MSS (Supplementary Table 3; see also the IHC results). One of the conceivable causes for this inconsistency regarding HC24T was the poor resolution of the electropherogram with the FFPE tissue-derived DNA as we described above, although the possibility of tumor microheterogeneity could not be excluded thoroughly.

Accordingly, the sensitivity (the MSI-H frequency by on-chip tests out of the cases diagnosed firmly by DNA sequencing and IHC analyses) and specificity (the frequency of the true MSI-H cases out of the cases designated as MSI-H by on-chip tests including false positives) of our on-chip MSI test with spheroid-derived DNA samples were both 100%, whereas those with the FFPE tissue-derived DNA was 60% and 98%, respectively. Thus, these results showed that spheroid-derived DNA samples provided more reliable data than FFPE tumor DNA.

To compare the quality of the DNA samples from the two sources further, we analyzed the MSI-H cases regarding the mononucleotide-repeat sequences of the four target genes that could be affected by MMR deficiency. The frameshift-mutation frequencies in the respective genes in spheroid-derived DNA were 100% for *TGFBR2*, 43% for *IGFR2*, and 86% for *BAX* and *CASP5* (Figure 5B). Notably, the frequencies detected with FFPE tissue-derived DNA samples were significantly lower than those with spheroid DNA (Figure 5B). It is likely that the compromised sensitivity of FFPE tumor DNA was caused by the stromal cells in cancer tissues. For example, the colon cancer HC49T had G7/G7 homozygous mutation of *BAX* in the spheroid-derived DNA (Figure 5C top). With the FFPE tumor DNA, however, peaks representing the wild-type stromal cells dominated, confused the interpretation, and led to misdiagnosis of this case as “wild-type” (Figure 5C bottom).

On the other hand, colon cancer HC106T was diagnosed as MSI-L by on-chip electrophoresis with FFPE tumor DNA. However, the spheroid DNA of the same tumor was diagnosed as MSI-H in seven-marker on-chip analysis because it had multiple peaks shifted from the normal cell DNA for *D2S123, BAT40* and *MYCL* as described above (Supplementary Figure 1). In addition, the IHC results showed lack of MLH1 and PMS2 in both the whole tumor and spheroids (data not shown). Consistently, direct sequencing of four MMR-target mononucleotide repeats showed that *TGFBR2* and *BAX* contained homozygous and heterozygous frameshift mutations, respectively, although their *IGF2R* and *CASP5* were homozygous wild type (Table 2). Mutational burden assessment also showed 61 and 9 total and indel mutations, respectively. Again, the discrepancy of the on-chip electrophoresis data between the DNA samples derived from spheroids and FFPE tumors are likely to be explained by the poor quality of DNA samples from FFPE tumors.

## DISCUSSION

The MMR-deficient colorectal cancer syndromes include mainly two types; Lynch syndrome (LS) that is caused by hereditary (i.e., familial) mutations in the MMR protein genes, and sporadic disease without familial background [1]. The latter can be caused either by somatic mutations or epigenetic inactivation of the MMR protein genes [17]. Diagnosis of Lynch syndrome patient could be performed by germline mutation analysis of MMR protein genes or *EpCAM* using DNA extracted from blood cells [24]. In this study, however, we did not distinguish these two subtypes. This is because both types of patients respond to immune checkpoint therapy very well likely because of the neoantigens generated by frameshift mutations in the coding regions of multiple genes [30]. Accordingly, it is worth the effort to maximize the diagnostic accuracy for identifying possible responders to the immune checkpoint therapy.

Notably, recent recommendations suggest that all colorectal cancer patients should be screened for MMR-deficiency first by IHC which is currently more economical than PCR-based DNA sequencing [6]. As we faced misdiagnosis (Supplementary Figure 6), however, IHC is not without pitfalls [29]. For example, it was reported that IHC of MMR proteins caused considerable errors; 10 of 14 cases assessed by IHC showed discrepant results with that of PCR analysis [29]. Besides, oxaliplatin-induced *MLH1* methylation and a specific mutation V384D in the same gene can give confusing results in IHC (Supplementary Figures 6 and 7). It is also worth noting that mutations in the DNA polymerase ε catalytic subunit gene (*DPOE1*) can cause hypermutational phenotype similar to MSI [18], although we found no such cases among 49 tested.

Here we evaluated multiple diagnostic measures using patient-derived TIC spheroid DNA and IHC, and demonstrated that the accuracy of MSI diagnosis was improved substantially. Because preparation of cancer spheroids from excised tumors has become less labor-intensive and more cost-effective [14], it appears worth the effort considering the very high therapeutic costs for immune checkpoint therapy.

With more advances in technology, mutational burden assessment may become cheaper in a near future [31]. Moreover, the pure and high quality cancer spheroid DNA can make the analysis scheme simpler. On the other hand, analyses of frozen cancer tissues, not to mention FFPE ones, need additional improvements to eliminate noises caused by non-cancer stromal cells. Because tumor mutational burden was distinctively high in MSI-H colorectal cancer when determined with spheroid DNA, it is possible practically to reduce the sequencing depth from the current > 500 repeats down to > 250 or less, as reported in a similar analysis [19, 21], which helps reduce the sequencing cost.

We have demonstrated that heterozygous mutations in the coding mononucleotide-repeats were detected unambiguously with cancer spheroid DNA, which cannot be attained with FFPE-tumor DNA. Although such heterozygous mutations do not cause loss of the gene functions usually, they can generate neoantigens that are targeted by the host immune system, which awaits further studies.

Some diagnostic markers are being implemented to stratify the cancer patients for immune checkpoint therapy. For example, expression of PD-1 ligands on cancer cell surface detected by IHC correlates with good response to the therapy [32]. Likewise, mutations in the 3’-untranslated region of the *PD-L1* gene affect its expression level through mRNA stabilization [33]. Accurate diagnosis of MMR-deficient colorectal cancer cases in combination with other functional markers should help maximize the patient benefits.

Here we have evaluated multiple diagnostic measures using spheroid-derived DNA and spheroid IHC. While the final recommendation of the diagnostic method can be made based on the disease stage and socioeconomic conditions of the patient, it is worth culturing and utilizing the TIC spheroids from the excised primary cancer, which helps improve diagnostic accuracy significantly.

## MATERIALS AND METHODS

### Human samples

A total of 111 human colorectal cancer samples were obtained from patients who underwent resection operations between January 2015 and October 2017 at Kyoto University Hospital (KUHP). The study protocol was approved by the institutional review board of Kyoto University, and written informed consents were obtained from the patients.

### Establishment and maintenance of patient-derived epithelial spheroid lines

Patient-derived spheroids of the colorectal cancer and normal colonic epithelium were established and maintained according to previous protocols [13, 14]. Ninety cancer spheroid lines from 89 patients and their genetic mutations were reported previously [14], whereas 21 lines from 21 patients were established thereafter in the present study.

### Preparation of spheroid DNA samples

Genomic DNA samples were extracted from normal and cancer epithelial spheroids cultured in 4 wells of 12-well cell-culture plates (∼10^5^ cells) according to the previous protocol [14].

### Genomic DNA preparation from formalin-fixed paraffin-embedded tissue sections

All cancer tissue sections were evaluated pathologically with H&E-stained slides to select tumor cell-enriched areas by a board-certified pathologist. Normal and cancer tissues were macrodissected with scalpel blades from at least three 10-µm FFPE tissue sections adjoining the H&E-stained section for each primary lesion. Dissected cancer tissue samples contained > 50% cancer cells. Normal colonic epithelium was collected from resected samples and their stem cells were cultured *in vitro* [14]. Genomic DNA was extracted using QIAamp DNA FFPE tissue kit (Qiagen, Hilden, Germany).

### On-chip microsatellite instability test

Microsatellite DNA fragments were amplified from spheroid DNA samples using Multiplex PCR Kit (Qiagen) and primer pairs for the Bethesda panel markers [2, 34] (Supplementary Table 4). Three electrophoretic runs were performed for each sample (*BAT25*/*D2S123, D5S346*/*D17S250*, and *BAT26*) according to the standard protocol [16] (Supplementary Table 5). Amplified DNA fragments were purified with QIAquick PCR Purification Kit (Qiagen), applied on the DNA LabChip of the Agilent DNA 1000 Kit (Agilent Technologies, Santa Clara, CA, USA), and analyzed with an Agilent 2100 Bioanalyser instrument (Agilent Technologies) according to the previous protocol [16]. The electropherogram of each cancer spheroid line was overlaid with that of the normal epithelial spheroid line derived from the same patient. A marker was diagnosed as unstable if any of the cancer peaks for the marker shifted more than 0.5 seconds. The MSI status of each case was determined as follows: MSI-high (MSI-H), with more than one unstable markers; MSI-low (MSI-L), with one unstable marker; and MSS, with no unstable markers. If only dinucleotide repeat markers as *D2S123*, *D5S346* and/or *D17S250* were mutated, a secondary panel of markers with mononucleotide repeats (*BAT40* and *MYCL*) was tested as recommended [1].

### Sequence analysis of mononucleotide repeats in cancer-related coding genes

The DNA fragments containing mononucleotide repeats in *TGFBR2* (A_10_), *BAX* (G_8_), *IGF2R* (G_8_), and *CASP5* (A_10_) were amplified from cancer spheroid DNA samples using JumpStart Taq DNA Polymerase (Sigma-Aldrich, St. Louis, MO, USA) or PrimeSTAR Max DNA Polymerase (Takara Bio, Kusatsu, Japan). Primer sequences and PCR conditions are shown in Supplementary Tables 4 and 5 [35]. Purified PCR fragments were sequenced by Macrogen (Seoul, Republic of Korea).

### Next generation sequencing

Next generation sequencing analyses of somatic cancer mutations were performed by Macrogen. In brief, DNA fragments that cover all exons of 409 cancer-related genes spanning a total length of 1.29 Mb (Ion AmpliSeq Comprehensive Cancer Panel; Thermo Fisher) were amplified from DNA samples of 7 MSI-H and 11 MSI-L or MSS cancer spheroid lines, and sequenced with the Ion Proton sequencer (Thermo Fisher).

### Estimation of mutational burdens (see Supplementary Figure 2)

The sequencing data were processed using Ion Torrent Suite Software v5.0.4 (Thermo Fisher), and variants against the hg19 human genome reference were called using Torrent Variant Caller v5.0.4 (Thermo Fisher). Possible cancer-specific mutations were selected through the following processes. The polymorphic alleles in 1,200 Japanese individuals were removed from each variant [36]. Only variants in the coding regions with > 20% frequency without polymorphism were scored. Erroneous mutations were eliminated by surveying their coverage tracks on Integrative Genomics Viewer software v2.3 (Broad Institute).

### Histological specimens

Formalin-fixed paraffin-embedded (FFPE) tumor specimens were prepared by the standard procedures in Department of Diagnostic Pathology, Kyoto University Hospital. A board-certificated pathologist selected multiple and separate cancer lesions from a single tumor. Specimens of FFPE cancer spheroids were prepared as previously reported [14]. These specimens were sectioned at 4-μm thickness, and stained with H&E or immunostained for MLH-1 (M1, Ventana, Tucson, AZ, USA), MSH2 (G219-1129, Ventana), MSH6 (EPR3945, Abcam, Cambridge, UK), or PMS2 (EPR 3947, Ventana) followed by hematoxylin counterstaining for nuclei.

### Agarose gel electrophoresis of genomic DNA

Genomic DNA samples from spheroids and FFPE tissues were electrophoresed on a 0.7% agarose gel. The gel was stained with ethidium bromide, and DNA fragments were visualized using Gel Doc XR+ (BioRad, Hercules, CA, USA) and Image Lab Software 3.0.1 Beta 2 (BioRad).

### Statistical analysis

Statistical analyses were conducted using GraphPad Prism 6 (Graph Pad Software, La Jolla, CA, USA). Mann-Whitney test was applied to compare DNA quality and electropherogram peak heights between spheroid- and FFPE-derived samples, and to compare total or indel mutational burden between MSI-H cases and MSI-L or MSS cases.

## SUPPLEMENTARY MATERIALS FIGURES AND TABLES







## Abbreviations

MMR: mismatch repair
MSI: microsatellite instability
PD-1: programmed cell death 1
PD-L1: programmed cell death 1 ligand 1
TIC: tumor-initiating cells
IHC: Immunohistochemistry
FFPE: formalin-fixed paraffin-embedded
MSI-H: microsatellite instability-high
MSI-L: microsatellite instability-low
MSS: microsatellite-stable
TGFBR2: transforming growth factor-beta receptor type 2
BAX: BCL2-associated X protein
IGF2R: insulin-like growth factor 2 receptor
CAS P5: caspase 5
WT: wild type
LS: Lynch syndrome
DPOE1: DNA polymerase ε catalytic subunit
CRC: colorectal cancer
MMRp: mismatch repair proficient
MMRd: mismatch repair deficient
