# Supplementary material for: Accurate diagnosis of mismatch repair deficiency in colorectal cancer using high-quality DNA samples from cultured stem cells
Source: Oncotarget. 2018 Dec 25;9(101):37534–48. doi: 10.18632/oncotarget.26495 (PMC6331025; doi:10.18632/oncotarget.26495)
Supplement: Supplementary file 1 [file oncotarget-09-37534-s001.pdf]

Accurate diagnosis of mismatch repair deficiency in colorectal cancer using high-quality DNA samples from cultured stem cells

SUPPLEMENTARY MATERIALS

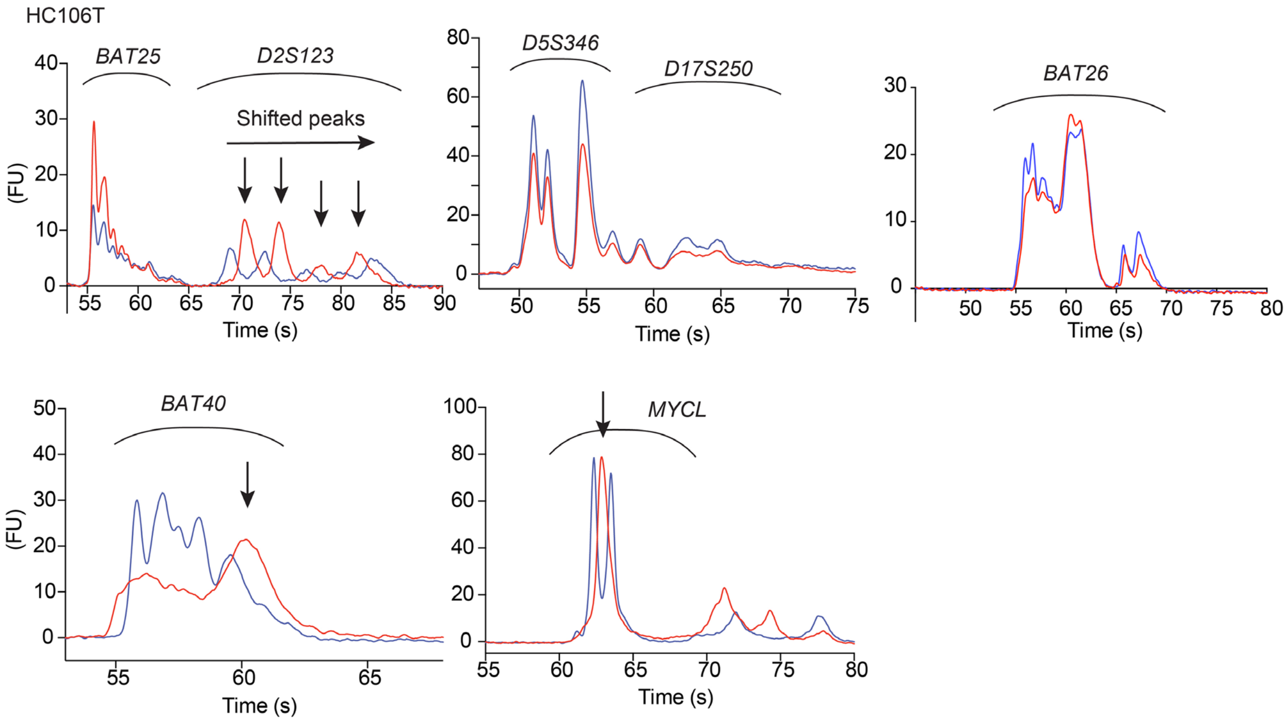

Supplementary Figure 1: Representative shifted peak patterns in electropherogram of cancer cell DNA samples from those of normal cells. In *D2S123*, the peaks for the cancer cells shifted to the right although the peak patterns between the normal (blue) and cancer cells (red) were similar. These shifted peaks reflect DNA fragment length changes caused by base-insertion mutations. Note that additional mononucleotide repeat markers *BAT40* and *MYCL* also showed shifted peaks in the cancer cell DNA sample.

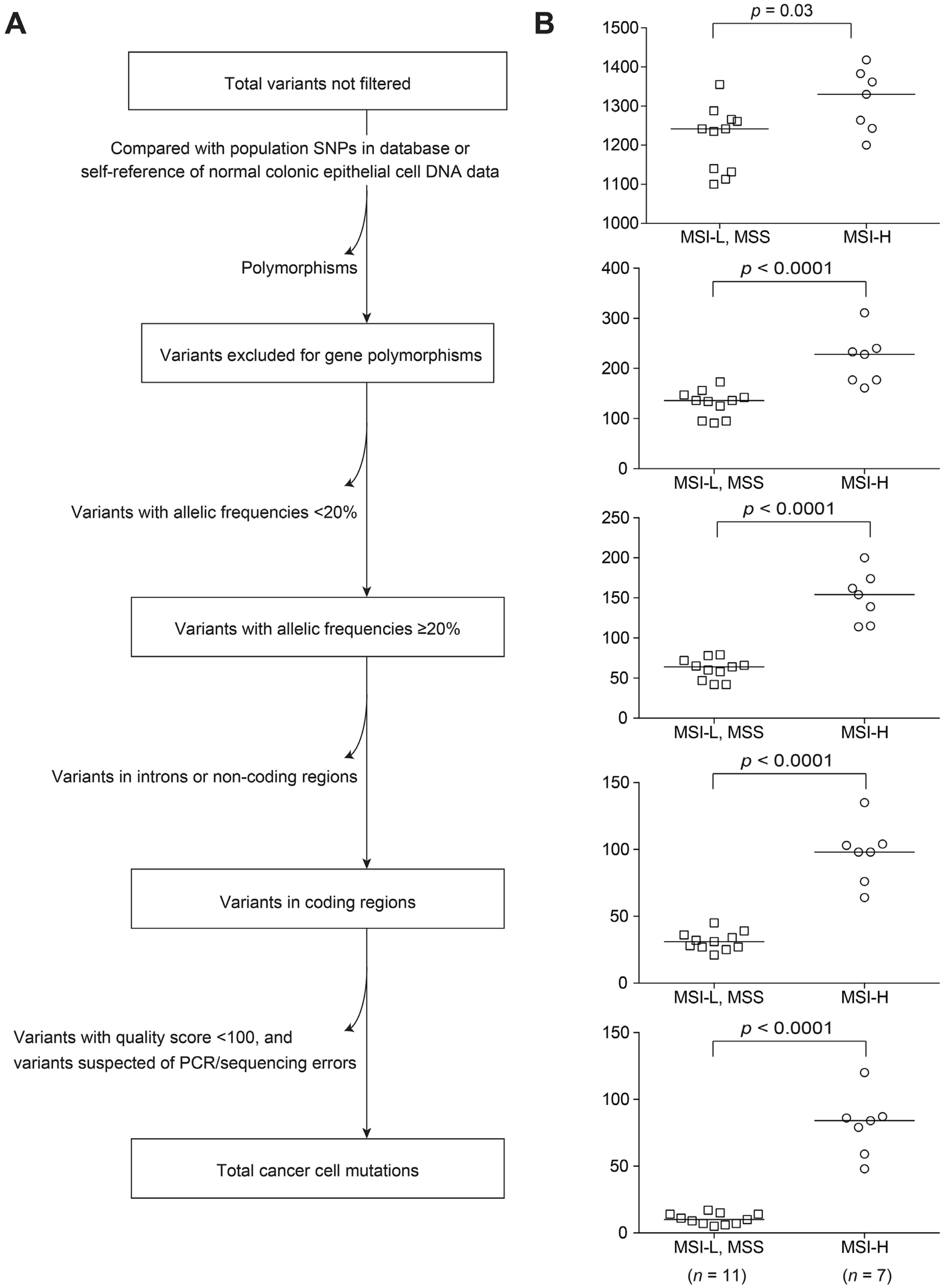

Supplementary Figure 2: Mutational burden assessment of NGS data through filtering of variants. (**A**) Filtering diagram for mutational burden estimation using the reference SNP database of a healthy local population (see Materials and Methods). (**B**) Effects of filtering at the respective steps on the apparent mutational burden compared between the MSI-L/MSS and MSI-H cases. Horizontal bars indicate the median values. The ordinate indicates the number of variants/mutations.

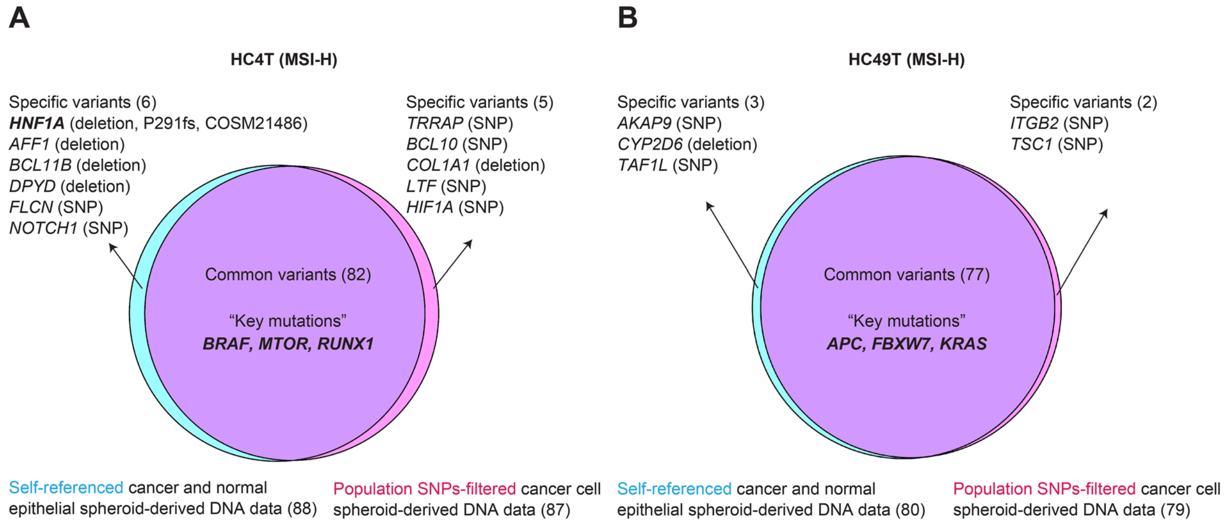

Supplementary Figure 3: Comparison of mutational burden data filtering methods between self-DNA referring and population-based SNP-database referring. (**A**, **B**) Two MSI-H colon cancer cases HC4T (A) and HC49T (B) were analyzed using two methods. In self DNA referring of NSG data using paired DNA samples of normal and cancer cells, variant data of the cancer spheroids were subtracted with those of the normal colonic epithelial spheroids from the same patients (left). Note that the mutations in the key cancer-related genes such as *BRAF* and *APC* were identical between the two methods. In the population-based SNPs method, variant data of cancer genome were subtracted with those of a healthy local population database (right).

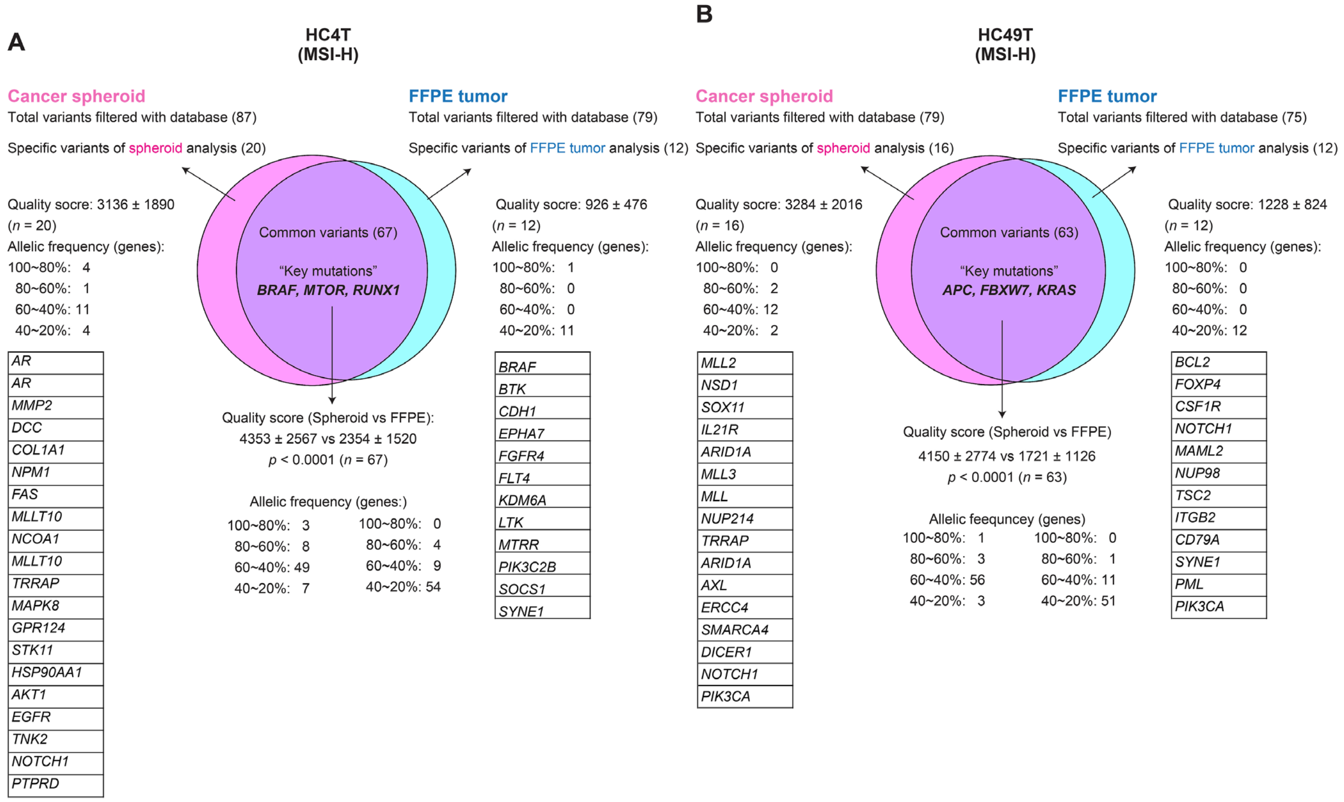

Supplementary Figure 4: Comparison of mutational burden data between the spheroid- and FFPE tumor-derived DNA samples. (**A**, **B**) NSG analysis of spheroid DNA (left) and FFPE tumor DNA (right) samples. Two MSI-H colon cancer cases HC4T (A) and HC49T (B) were analyzed as in Supplementary Figure 3 above. Note the significantly higher quality score of variants with spheroid-derived DNA samples than FFPE-tumor derived ones, and that the allelic frequency was also significantly higher with the spheroid-derived DNA. The mutations in the key cancer-related genes such as *BRAF* (V600E) and *APC* were identical between the two sources, and more numbers of variants were found in the spheroid DNA samples than in the FFPE tumor samples. An additional *BRAF* mutation A29fs was detected in HC4T FFPE tumor.

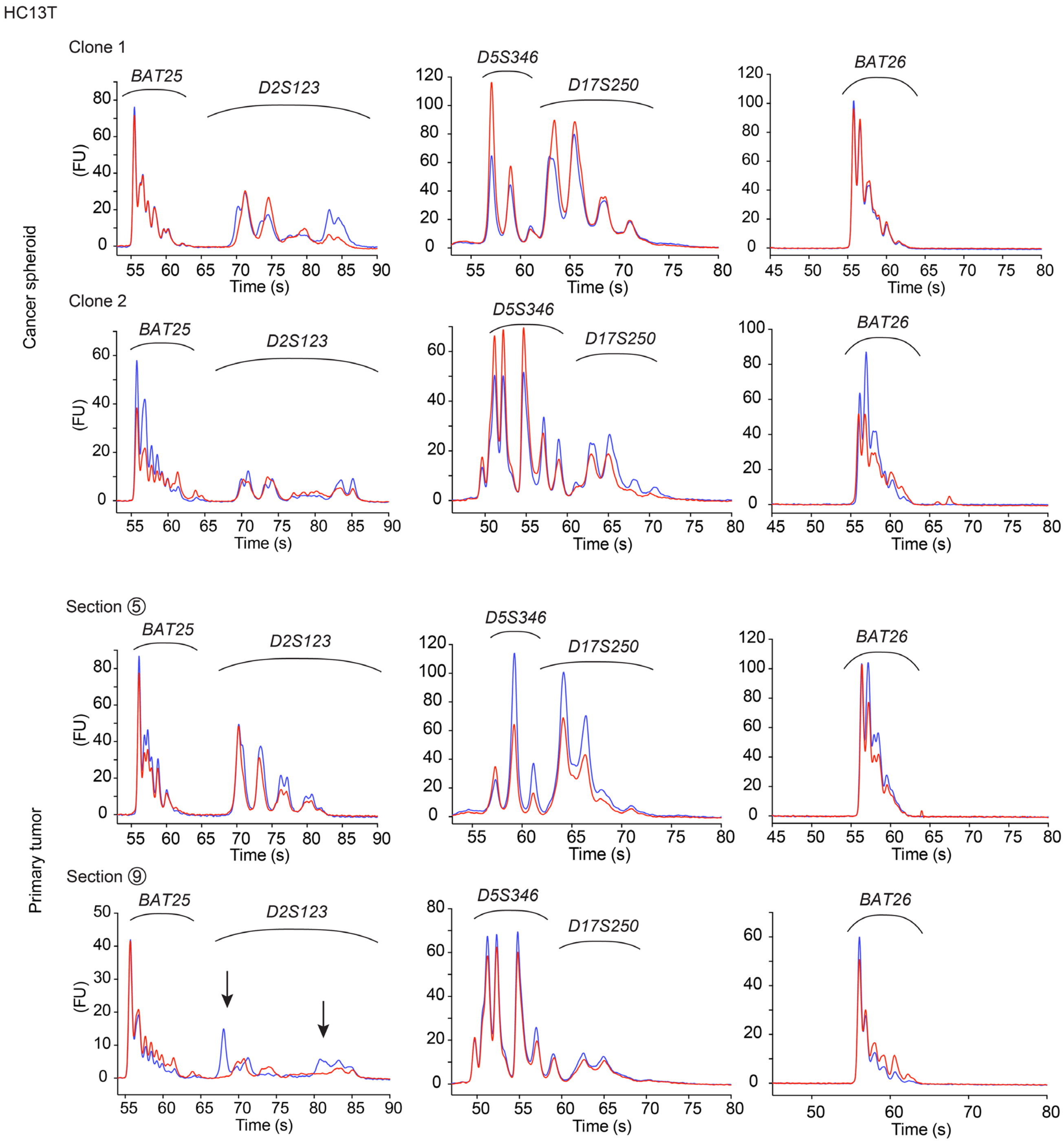

Supplementary Figure 5: On-chip analysis of colon cancer HC13T DNA samples extracted from its spheroid cell lines and from FFPE tumor tissues. For both independently isolated cancer spheroid lines (Clone 1 and Clone 2) isolated from this particular case HC13T, the on-chip electrophoresis of MMR target markers showed identical patterns to their normal epithelial spheroids. Likewise, DNA samples extracted from two separate sub-lesions of the primary tumor (Section 5 and 9; See Supplementary Figure 6) showed MSS and MSI-L.

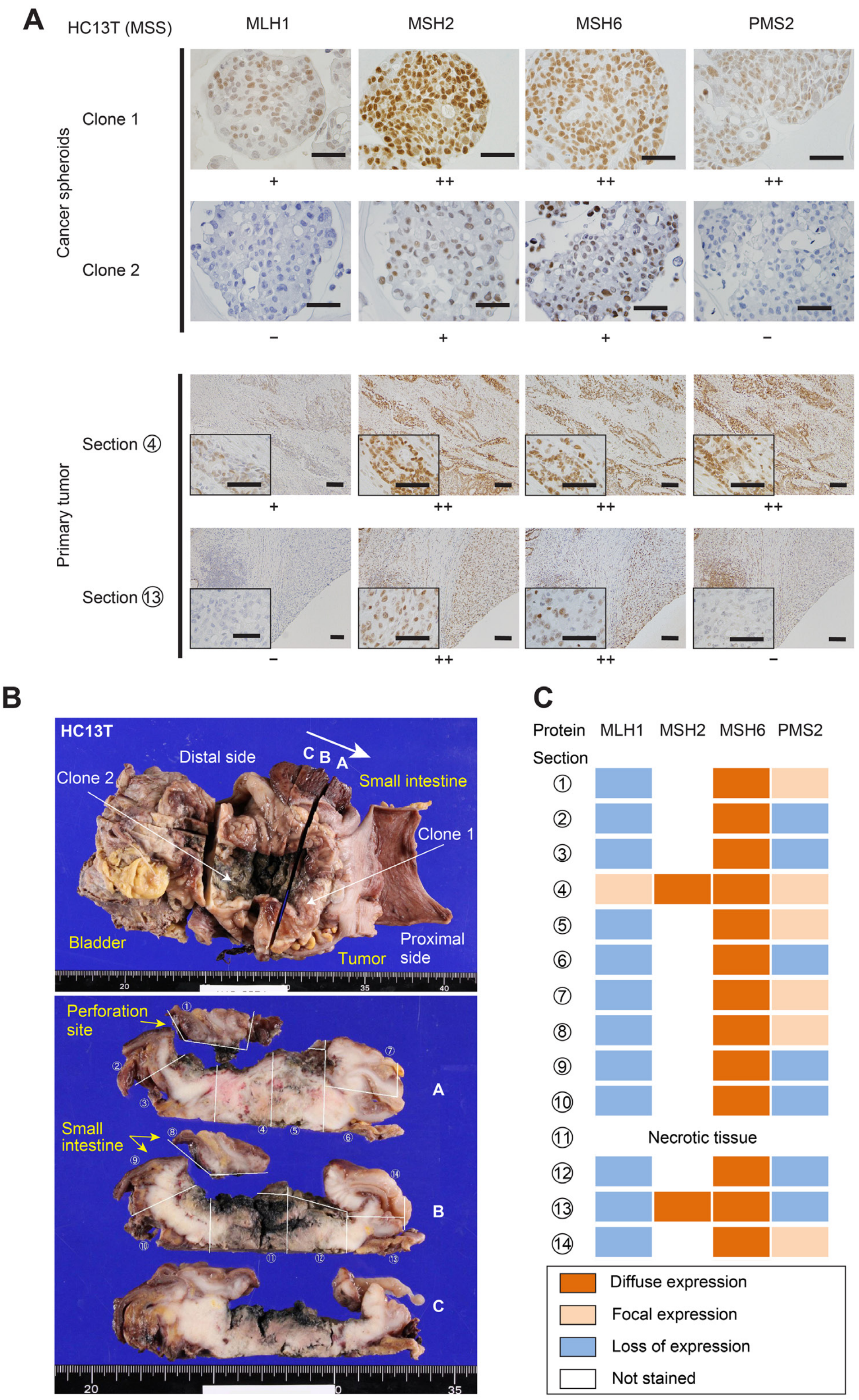

Supplementary Figure 6: Immunohistochemistry of a colon cancer HC13T that showed different results between DNA analyses and IHC of MMR proteins. (**A**) Upon IHC of the two independent spheroid lines of HC13T (see Supplementary Figure S5 above), they showed discrepant results each other. Although FFPE spheroids of Clone 1 was stained for all four MMR proteins, those of Clone 2 lacked staining for MLH1 and PMS2 (top rows). Likewise, sub-lesions of the primary tumor sections also showed different staining results for the MMR proteins. For example, Section 4 had all four MMR proteins, whereas Section 13 lacked MLH1 and PMS2 (bottom rows). (**B**) Macroscopic views of colon cancer case HC13T. Luminal view of the tumor containing colon in the rostro-caudal orientation (top), and three cross-sectional views at A, B, and C shown in top view (bottom). Sections obtained for IHC are indicated by encircled numbers. (**C**) Schematic summary of the IHC results for three MMR proteins, MLH1, MSH6, and PMS2 for 14 sub-lesions shown in B. As a reference, MSH2 was also stained for two sections (Sections 4 and 13). Note that Section 11 was necrotic and unanalyzable.

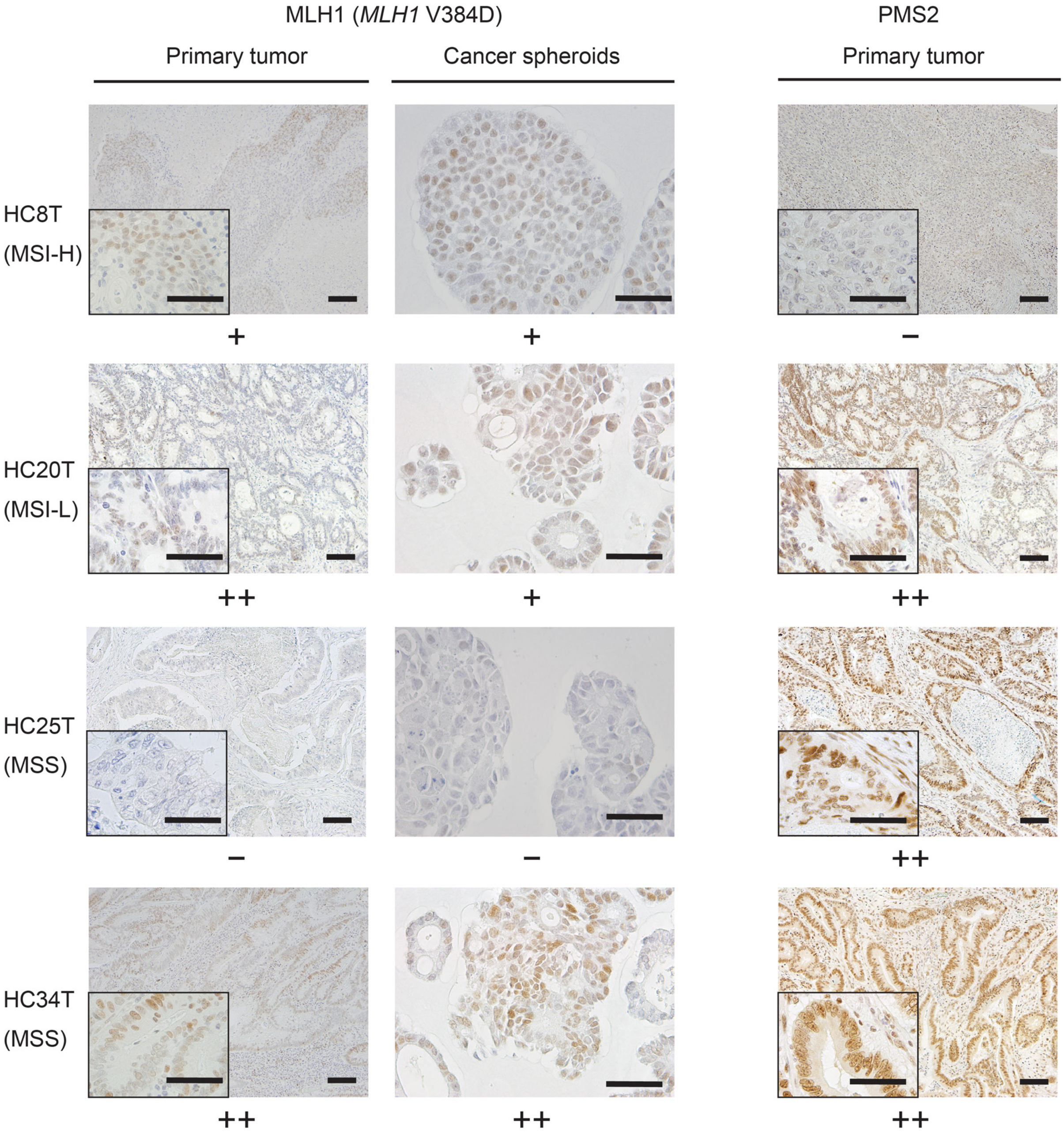

Supplementary Figure 7: IHC analysis of four MLH1 V384D mutant colon cancer cells for MLH1 and PMS2 proteins. Apparent changes in MLH1 immunoreactivity in some of the MLH1 V384D mutation-carrying colon cancer cells. Although HC8T stained positive for MLH1, it was negative for PMS2 (top row), and its DNA analyses indicated MSI-H (Table 2 and Figure 5B). On the other hand, HC25T tumor was positively stained for PMS2 despite its negative staining for MLH1 by IHC (third row). It was diagnosed as MSS by on-chip satellite marker DNA analysis. In two other cases (HC20T and HC34T; second and fourth rows, respectively), both MLH1 and PMS2 were detected by IHC, and DNA analyses indicated MSI-L and MSS (data not shown), despite carrying the V384D mutation of MLH1.

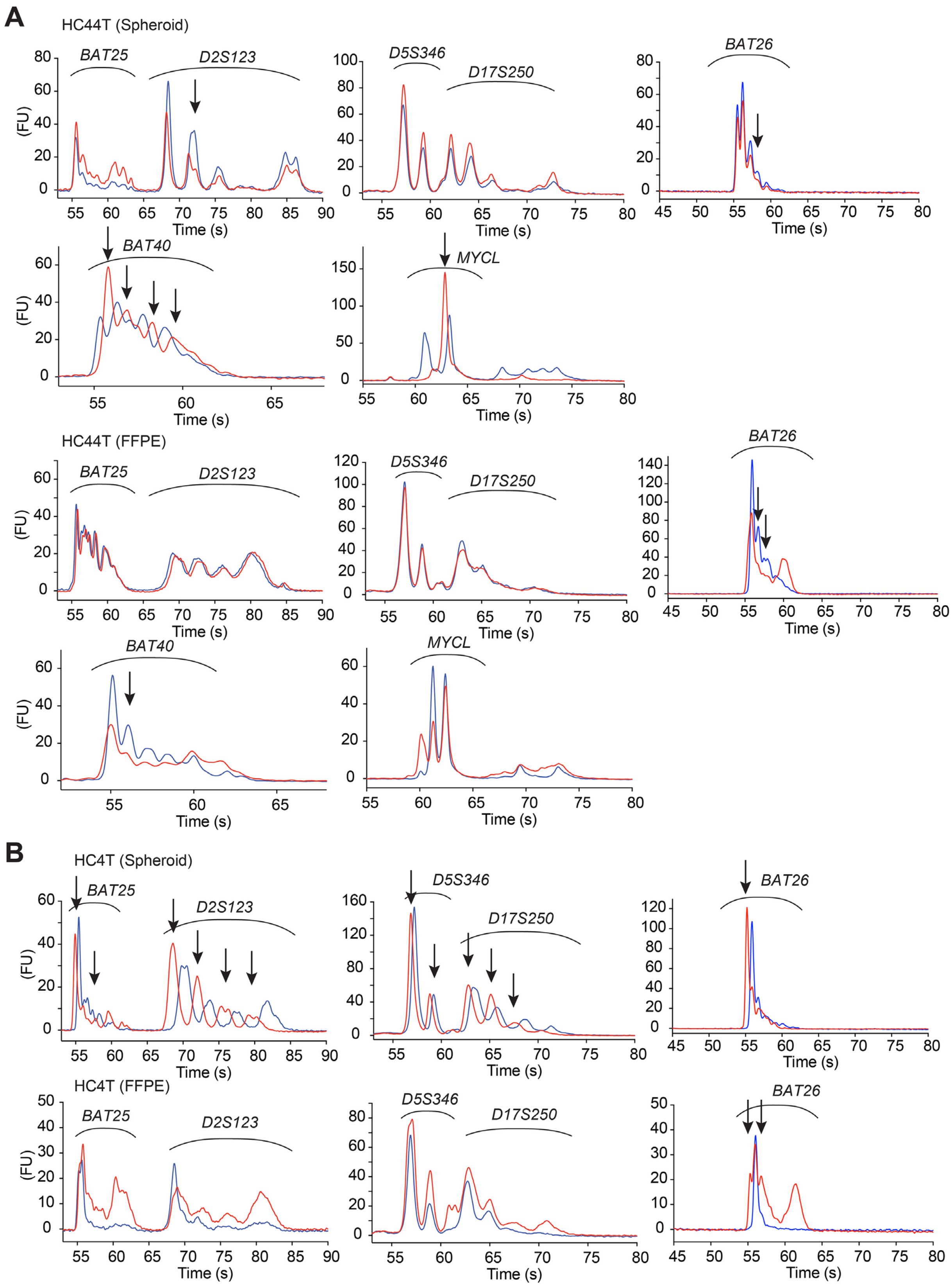

Supplementary Figure 8: On-chip electropherograms of MSI markers amplified from colon cancer cases that showed discrepant results between spheroid- and FFPE tumor-derived DNA samples. Discrepancies were found in two false-negative cases (Supplementary Figure 8, (**A**) HC44T and (**B**) HC4T) and one false-positive (Supplementary Figure 9; HC24T). For HC44T, the results with *BAT26* were difficult to be evaluated. Accordingly, additional markers were tested, leading to the final diagnosis of MSI-H. On the other hand, PCR amplifications of *BAT40* and *MYCL* on FFPE tumor-derived DNA samples of HC4T failed to produce analyzable-quality products, and therefore not shown in Supplementary Figure 8.

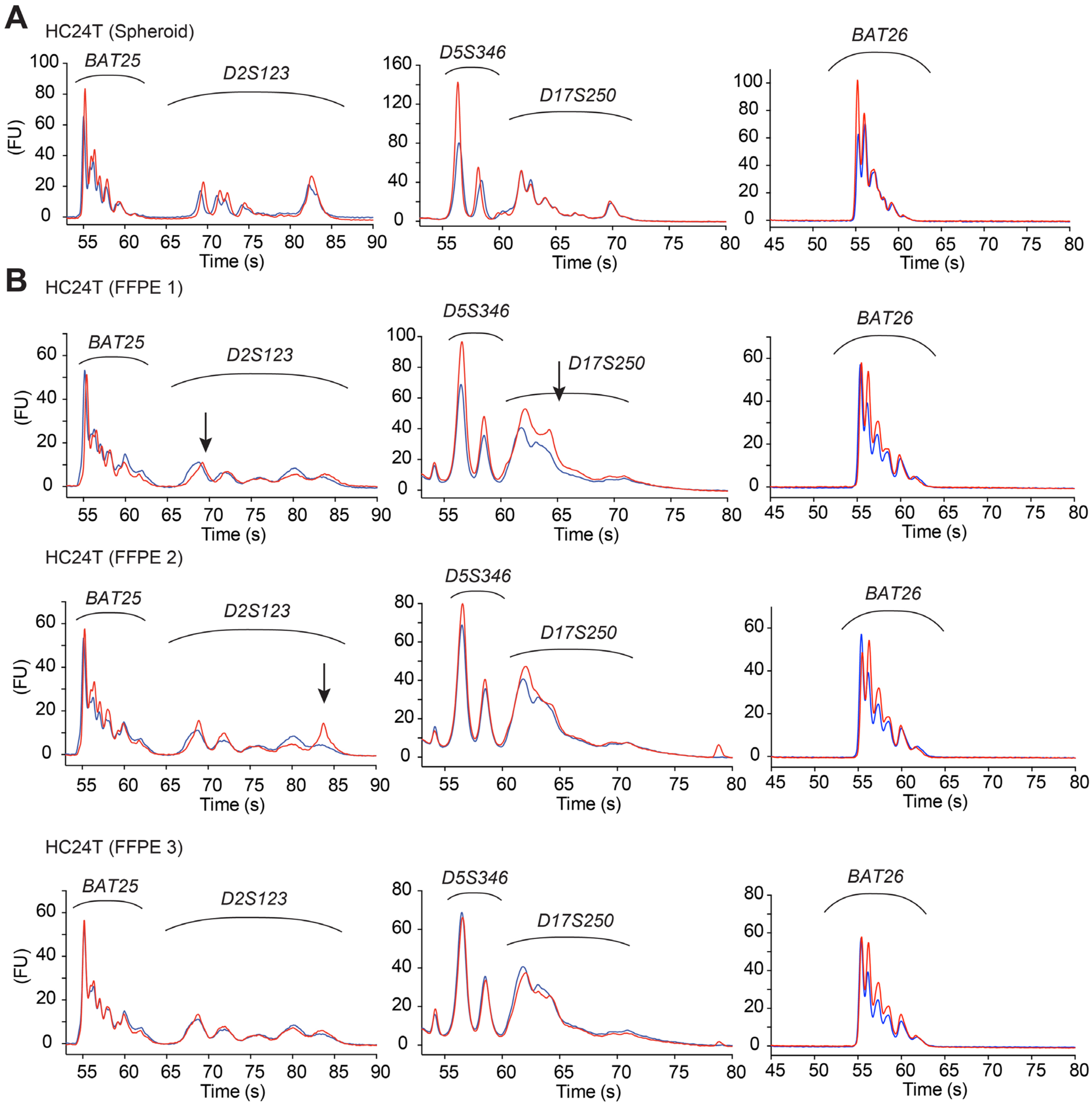

Supplementary Figure 9: On-chip electropherograms of MSI markers amplified from colon cancer cases that showed discrepant results between spheroid- and FFPE tumor-derived DNA samples. Discrepancies were found in two false-negative cases (Supplementary Figure 8) and one false-positive (Supplementary Figure 9; HC24T).

Supplementary Table 1: Potential cancer-driving mutation list of all seven MSI-H cases. See Supplementary_Table_1

Supplementary Table 2: Potential cancer-driving mutation list of 11 MSI-L/MSS cases. See Supplementary_Table_2

Supplementary Table 3: Colorectal cancer cases that produced discrepant results between different DNA sources or IHC specimens

Method	On-chip		Sequencing of MMR deficiency target genes[c]				IHC	
**Case**	**MSI status[a] (Sph$_b$)**	**MSI status[a] (FFPE)**	***TGFBR2*** **(WT: A_{10})**	***IGF2R*** **(WT: G_8)**	***BAX*** **(WT: G_8)**	***CASP5*** **(WT: A_{10})**	**Absent proteins[d] (Sph[b])**	**Absent proteins[d] (Primary)**
HC13T[e]								
Spheroid Clone 1	MSS		WT/WT	WT/WT	WT/WT	WT/WT	None	
Spheroid Clone 2	MSS		WT/WT	WT/WT	WT/WT	WT/WT	MLH1, PMS2	
FFPE 1 Sec. 5[f]		MSS	WT		WT			MLH1
FFPE 2 Sec. 9[f]		MSI-L	WT		WT			MLH1, PMS2
HC44T	H	L	WT/A_{11}	WT/WT	WT/G_9	WT/A_9	MLH1, PMS2	MLH1, PMS2
HC4T	H	L	A_9/A_9	WT/G_7	G_7/G_7	A_9/A_9	MLH1, PMS2	MLH1, PMS2
HC24T	MSS	H	WT/WT	WT/WT	WT/WT	WT/WT	None	None
HC106T[g]	H	L	A_8/A_9	WT/WT	WT/G_9	WT/WT	MLH1, PMS2	MLH1, PMS2

[a]H: MSI-High, L: MSI-Low
[b]Spheroid
[c]Only spheroid DNA data are shown, except in HC13T.
[d]Absent MMR proteins in immunohistochemical staining
[e]This case with the discrepant results between genetic analyses and IHC for MMR proteins.
[f]FFPE tissue sections of primary tumor (Supplementary Figure 6).
[g]This tumor was not included in the initial set of 50 cases of which DNA samples extracted from spheroids and FFPE tumors were compared.

Supplementary Table 4: PCR primers used in this study

Marker	Primer pair (5′ to 3′)	Reference
BAT25	TCGCCTCCAAGAATGTAAGT	[34]
	TCTGCATTTTAACTATGGCTC	
D2S123	AAACAGGATGCCTGCCTTTA	[34]
	GGACTTTCCACCTATGGGAC	
D5S346	ACTCACTCTAGTGATAAATCG	[34]
	AGCAGATAAGACAGTATTACTAGTT	
D17S250	GGAAGAATCAAATAGACAAT	[34]
	GCTGGCCATATATATATTTAAACC	
BAT26	TGACTACTTTTGACTTCAGCC	[34]
	AACCATTCAACATTTTTAACCC	
BAT40	ATTAACTTCCTACACCACAAC	[34]
	GTAGAGCAAGACCACCTTG	
MYCL1	TGGCGAGACTCCATCAAAG	[34]
	CTTTTTAAGCTGCAACAATTTC	
TGFBR2	TCCAATGAAT CTCTTCACTC	[35]
	CCCACACCCT TAAGAGAAGA	
IGF2R	CAGGTCTCCT GACTCAGAAG	This study
	CTAATATGATCCCAGCAGCC	
BAX	GGCTGCTGG GATCATATTAG	This study
	CCTCTGCAGC TCCATGTTAC	
CASP5	GTGTTATTCGCTGGAGACATGG	This study
	CAAGATCAGGGCCTTGTCTTC	

Supplementary Table 5: PCR conditions used in this study

Marker	Enzyme	Initial denaturation	Amplification	Finial elongation
BAT25/D2S123	M[a]	95°C (15 min)	94°C (30 sec) – 60°C (90 sec) – 72°C (60 sec) 35 cycles	60°C (30 min)
D5S346/D17S250	M[a]	95°C (15 min)	94°C (30 sec) – 50°C (90 sec) – 72°C (60 sec) 35 cycles	60°C (30 min)
BAT26	M[a]	95°C (15 min)	94°C (30 sec) – 60°C (90 sec) – 72°C (60 sec) 35 cycles	60°C (30 min)
BAT40 *MYCL1*	M[a]	95°C (15 min)	94°C (30 sec) – 55°C (90 sec) – 72°C (60 sec) 35 cycles	60°C (30 min)
TGFBR2	P[b]	95°C (3 min)	98°C (10 sec) – 60°C (5 sec) – 72°C (30 sec) 35 cycles	60°C (10 min)
	J[c]	95°C (1 min)	94°C (30 sec) – 50°C (30 sec) – 72°C (30 sec) 35 cycles	72°C (10 min)
IGF2R *BAX* *CASP5*	J[c]	95°C (1 min)	94°C (30 sec) – 65°C (30 sec) – 72°C (30 sec) 35 cycles	72°C (10 min)

[a]Multiplex PCR Kit (Qiagen)
[b]PrimeSTAR Max DNA Polymerase (Takara)
[c]JumpStart Taq DNA Polymerase (Sigma)
